# Validity of the two different scoring methods of the Clinical Dementia Rating scale in staging and detection of cognitive impairment in the Peruvian population

**DOI:** 10.1002/alz.71550

**Published:** 2026-06-03

**Authors:** Junior Senador, Rosa Montesinos, Belén Custodio, Jose Huilca, Katherine Agüero, Graciet Verastegui, Pamela Bartolo, Milagros Nuñez‐Huanca, María Fe Albujar Pereira, Nilton Custodio

**Affiliations:** ^1^ Unidad de Investigación de Deterioro Cognitivo y Prevención de Demencia Instituto Peruano de Neurociencias Lima Peru; ^2^ Unidad de Investigación y Docencia Equilibria Lima Peru; ^3^ Centro de Investigación del Envejecimiento Universidad de San Martín de Porres Lima Peru

**Keywords:** Alzheimer's disease, Clinical Dementia Rating Scale Global Score, Clinical Dementia Rating Scale Sum of Boxes, dementia, mild cognitive impairment, Peruvian

## Abstract

**INTRODUCTION:**

In Peru, the Global Deterioration Scale is the only available tool for assessing dementia severity. The Spanish translation and cultural adaptation of the Clinical Dementia Rating (CDR) is used locally but requires formal psychometric validation in the Peruvian sociocultural context.

**METHODS:**

A total of 1,600 older adults: 902 controls, 271 mild cognitive impairment (MCI), and 427 Alzheimer's disease patients (AD) were assessed to validate two CDR scoring methods: CDR Global Score (CDR‐GS) and CDR Sum of Boxes Score (CDR‐SB). Discriminative validity, inter‐item reliability, and concurrent validity were evaluated using MMSE, RUDAS, PFAQ, and Uniform Data Set (UDS) v3 Neuropsychological Battery.

**RESULTS:**

CDR demonstrated high reliability and validity among normal cognition, mild cognitive impairment, and AD. For AD screening, CDR‐SB showed 98.2% sensitivity and 99.6% specificity, while CDR‐GS demonstrated 99.8% sensitivity and 100% specificity.

**DISCUSSION:**

The Peruvian CDR version exhibited good psychometric properties comparable to those reported in Latin American CDR validation studies.

## Highlights

- Two different scoring methods of Clinical Dementia Rating (CDR) showed high reliability and validity in distinguishing normal cognition, mild cognitive impairment (MCI), and Alzheimer's disease (AD) dementia.
- For AD screening, CDR Sum of Boxes (CDR‐SB) showed 98.2% sensitivity and 99.6% specificity, while CDR Global Score (CDR‐GS) demonstrated 99.8% sensitivity and 100% specificity.
- This study provides a basis for comparison with other CDR‐based studies in populations worldwide.

## 1 BACKGROUND

The progression of neurocognitive disorders represents a global challenge, leading to a growing demand for cognitive assessment to stage neurocognitive decline. The ability to detect clinical variations and changes within the dementia spectrum is of utmost relevance and essential for the design of future trials of disease‐modifying therapies.^1^ The Clinical Dementia Rating (CDR) scale^2^ is an used instrument in Alzheimer's disease (AD) for rating disease staging and monitoring intra‐individual changes, being an ideal candidate for adults with cognitive complaints, particularly in Latin America (LA). However, it has not yet undergone formal validation in Peru.

International diagnostic standards for AD, primarily established by Diagnostic and Statistical Manual of Mental Disorders Fifth Revision (DSM‐5)^3^ and the National Institute on Aging‐Alzheimer's Association (NIA‐AA),^4^ mandate a multi‐component approach based on biological aspects detected by specific biomarkers measured by positron emission tomography (PET), cerebrospinal fluid (CSF), or blood. Therefore, dementia's specialists need to harmonize the new diagnostic criteria based on local availability of biomarkers for LA, where PET‐ and CSF‐based biomarkers may not be readily available.^5^ To adapt the AT(N) framework, researchers from Brazil,^6^ Peru,^7^ and the ReDLat consortium^8^ have been demonstrating the potential utility of blood‐based biomarkers (BBB) for AD diagnosis. However, current evidence is derived exclusively from specialized research centers, highlighting the urgent need to validate robust clinical staging tools such as CDR for use in settings where biomarker access remains limited.

The dementia's diagnosis is clinical, based on clinical, cognitive and functional assessments. Brief cognitive and functional screening tools (BCSs and BFSs) can substantially improve the accuracy and timeliness of diagnoses^9^ supported by a neuropsychological assessment.^10^ In Peru validated BCSs and BFSs tools have been published for detecting dementia across diverse educational levels.^10^ The Uniform Data Set (UDS) from the National Alzheimer's Coordinating Center (NACC), with the Neuropsychological Battery measures domain‐specific and enabling precise characterization of cognitive impairment, was published.^11^ To assess dementia severity, there are only validations of the Global Deterioration Scale (GDS).^12^

The CDR evaluate cognitive function and assesses autonomy across six domains and usually assigns a score (0 for healthy, 0.5 for MCI, and 1, 2, and 3 for mild, moderate, and severe AD),^13^ enabling clear discrimination between healthy subjects and pathological stages.^14^ Quantifying functional decline and the severity of clinical impairment is critical for monitoring patients over time^15^ and in this sense, summing the ratings for each domain (or “box”) provides the quantitative Sum of Box score (CDR‐SB), with a range of 0 (no impairment) to 18 (severe impairment).^16^ The CDR training and certification protocols are available at https://knightadrc.wustl.edu/CDR/CDR.htm.^17^ Actually, CDR was extended for frontotemporal dementia (FTD) introducing a behaviour and a language domain,^18^ classifying cases into five severity levels based on the number and severity of the ratings given for the eight domains.^19^ Therefore, has shown the ability to detect mild to severe symptoms in sporadic and genetic FTD cohorts^19^ and capture disease progression over 1–2 years.^20^ But CDR is an ecological approach involving both the patient and the informant,^21^ whom does not require reading and writing skills, which is ideal in the Peruvian context, where educational gaps and urban‐rural disparities are prominent. However, future studies must extend beyond urban centers to include rural and low‐literacy populations, ensuring that diagnostic tools are representative of the region's full demographic spectrum.^22^ The present study aims to formally validate the CDR within the clinic‐based, urban, and middle‐level education Peruvian population, implying the evaluation of its two primary scoring methods: the Global Score (CDR‐GS; 0–3 ordinal staging of dementia severity) and the CDR‐SB (0–18 continuous measure of cognitive/functional impairment), offering complementary staging and sensitivity for MCI to dementia progression. The secondary objectives include: (1) to assess the reliability; (2) to determine the discriminative validity; and (3) evaluate the convergent validity between the CDR and other tests.

## 2 METHODS

### 2.1 Study design and participants

We conducted an observational, cross‐sectional study at the Research Department of the Instituto Peruano de Neurociencias (IPN). Participants (control: 902, MCI: 271, AD: 427) were enrolled consecutively between May 2020 and March 2025. Eligibility criteria included: (1) an established classification as a control participant (cognitively healthy) or diagnosis of neurocognitive disorder,^23^ mild cognitive impairment (MCI),^24^ or dementia due to Alzheimer's‐AD^4^ based on established clinical criteria; (2) presence of a formal or informal informant/caregiver (see below); (3) over 50 years of age; (4) caregiver ability to complete the questionnaire; (5) Spanish speaking as a primary language; and (6) had a minimum of 4 years of formal education. Caregivers were family members or close friends in direct contact with the patient for more than 6 hours each day for at least 3 consecutive months prior to the interview. Caregivers were family members or close friends who had direct contact with the patient for more than 6 hours each day for 3 consecutive months prior to the interview. Caregivers were also screened with the Zarit scale^25^ and Patient Health Questionnaire 9^26^ to exclude individuals with caregiver burnout (Zarit > 30) or depression (score > 4). Patients whose cognitive function could be impaired by the use of specific drugs or by a particular medical condition, including a history of addiction and substance abuse, depression, hypothyroidism, vitamin B12 deficiency, chronic kidney or liver disease, human immunodeficiency virus (HIV) or syphilis neuro‐infections, severe head trauma, and subdural hematoma, were excluded. Additionally, individuals who couldn't complete the UDS‐NB3.0 due to severe dementia or with conditions that could impact their cognitive performance during the evaluation were excluded: hearing or visual impairments; motor sequelae of cerebrovascular disorders; or traumatic sequelae.

### 2.2 Diagnostic process and final classification

The evaluations were conducted at the IPN research unit during standardized cognitive sessions (see *Neurocognitive and Functional Assessment*) for people with MCI and dementia, during which each participant underwent clinical and neuropsychological assessment. The control group comprised cognitively healthy individuals presenting at the clinic with cognitive complaints but had negative results on brief cognitive and functional tests. They underwent the same neurocognitive and functional evaluation as the case group. During these same sessions, the patients and informants were interviewed to complete the CDR.

During cognitive evaluation, each patient is subjected to a process of 3 successive phases: (1) screening, to detect cognitive impairment; (2) disease classification, diagnosis to rule out other causes of dementia; and (3) final classification, dementia subtype and severity of the disease. In the screening phase, we used validated Peruvian versions of brief cognitive and functional tests: Rowland Universal Dementia Assessment Scale (RUDAS)^27^ and Pfeffer Functional Activities Questionnaire (PFAQ).^28^ When the participant was classified as having cognitive impairment, they move on to the second phase to be evaluated with selected blood tests (hemogram, glucose, transaminase, urea, creatinine, vitamin B12, folic acid, free T3 and free T4, ultrasensitive thyroid stimulating hormone, Rapid Plasma Reagin (RPR) test, and an enzyme‐linked immunosorbent assay for HIV), brain magnetic resonance imaging (MRI), and Beck Depression Inventory II (BDI‐II) to rule out non‐degenerative causes of cognitive decline. Finally, each case of dementia was referred to the research unit to complete a full neuropsychological evaluation using the UDS‐Neuropsychological Battery from the NACC (UDS‐NB 3.0) which assesses episodic memory, processing speed, executive function, language, and visuo‐construction ability.^29^

**RESEARCH IN CONTEXT**

1. **Systematic review**: To date, several brief cognitive and functional assessments have been validated in Peru to detect mild cognitive impairment and dementia across diverse educational backgrounds. However, regarding the assessment of dementia severity, the only identified validation is of the Global Deterioration Scale. While a culturally adapted Spanish version of the Clinical Dementia Rating (CDR) is used locally, a formal psychometric validation within the Peruvian sociocultural context remains necessary.
2. **Interpretation**: The study demonstrated the clinical validity of the CDR for classifying dementia severity in a Peruvian population, establishing that the Spanish version may be appropriately applied for research in this setting, and provided evidence for two different scoring methods.
3. **Future directions**: Future work could examine the performance of the Peruvian CDR in primary care and community samples, as well as its longitudinal responsiveness to clinically meaningful change in mild cognitive impairment (MCI) and early Alzheimer's disease (AD).

**FIGURE 1:**
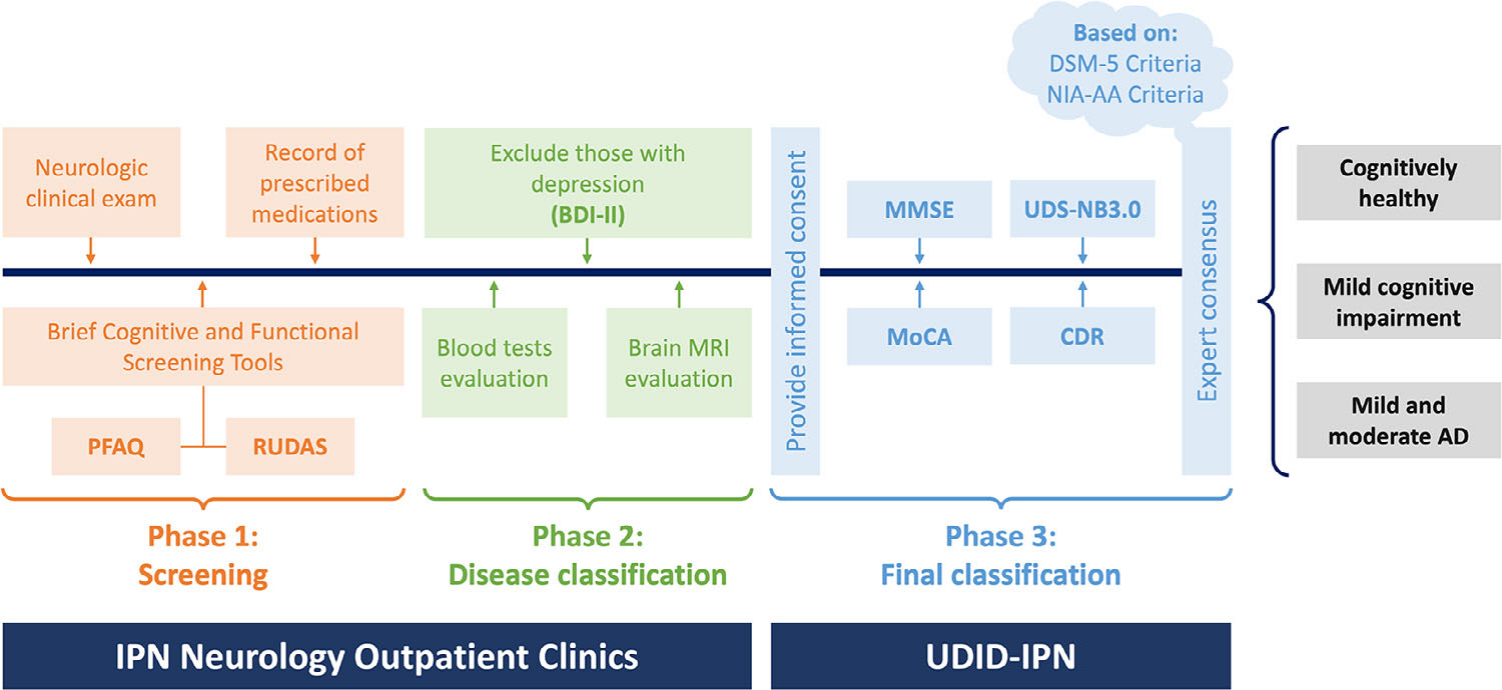
Flowchart of the diagnostic process of participants, cognitively healthy and patients with mild cognitive impairment and Alzheimer's dementia, to validate the two different scoring methods of the Clinical Dementia Rating scale in the Peruvian population. BDI‐II, Beck Depression Inventory‐II; CDR, Clinical Dementia Rating; DSM‐5, Diagnostic and Statistical Manual of Mental Disorders, Fifth Edition; IPN, Instituto Peruano de Neurociencias; MRI, magnetic resonance imaging; MMSE, Mini‐Mental State Examination; MoCA: Montreal cognitive assessment; NIA‐AA, National Institute on Aging‐Alzheimer's Association; PFAQ, Pfeffer Functional Activities Questionnaire; RUDAS, Rowland Universal Dementia Assessment Scale; UDID‐IPN, Unidad de Investigación y Docencia, Instituto Peruano de Neurociencias; UDS‐NB3.0, Uniform Data Set‐Neuropsychological Battery from the National Alzheimer's Coordinating Center's NACC.

After a consensus meeting among neurologists, geriatricians, psychiatrists, and neuropsychologists, the probable type of dementia was defined by applying the diagnostic criteria of DSM‐5 and NIA‐AA.^23^ The time interval between the final classification phase (including the administration of the CDR) and the diagnostic consensus was no more than 2 weeks (see Figure 1).

### 2.3 Neurocognitive assessment

#### 2.3.1 Mini‐Mental State Examination

We used the Peruvian adaptation of the Mini‐Mental State Examination (MMSE).^30^ The MMSE is a classical instrument for assessing cognitive domains, such as orientation (10 points), registration (3 points), attention and calculation (5 points), recall (3 points), and language and praxis (9 points). A score below 24 points has a sensitivity above 64% and a specificity close to 82% for detecting cognitive impairment in Peruvian patients with dementia.^31^

#### 2.3.2 RUDAS

The RUDAS can be administered within 10 minutes and comprises 6 sections with a maximum of 30 points: memory (8 points), visuospatial orientation (5 points), motor praxis (2 points), visuospatial construction (3 points), judgment (4 points), and semantic fluency (8 points). The RUDAS has a maximum score of 30, with lower scores indicating poorer cognitive performance. Several studies have been published validating the RUDAS among Peruvians with a middle‐education level from an urban area of Peru^32^ and urban^22^ and rural^33^ Peruvians with illiteracy. The most common cutoff for RUDAS is < 22, with reported sensitivity ranging from 0.78 to 0.86 and specificity from 0.78 to 0.87.^34^

#### 2.3.3 Montreal Cognitive Assessment

The Montreal Cognitive Assessment (MoCA) measures eight cognitive domains, which are scored within a range of 0–30 points (higher scores indicating better function): short‐term memory (delayed recall, 5 points); visuospatial abilities (cube drawing, 1 point, clock drawing, 3 points); executive function (Trail Making Test [TMT], 1 point; phonemic verbal fluency, 1 point; verbal abstraction, 2 points); attention, concentration, and working memory (cancellation, 1 point; subtraction, 3 points; digit span, 2 points); language (naming, 3 points; sentence repetition, 2 points); and orientation to time (3 points) and space 3 points). To correct for educational effects found in the original study, an additional point was given to subjects with 12 or fewer years of education, following the authors’ instructions and the procedure adopted by previous studies.^35^ We used the Spanish version 7.0 of MoCA and estimated total score and domain indices, according to the ReDLat protocol.^36^

### 2.4 Functional assessment

#### 2.4.1 PFAQ

PFAQ comprises 11 items assessing instrumental activities of daily living (I‐ADL). Each item is rated on a 4‐point scale from 0 (normal or never did but could do now), 1 (has difficulty but does by self or never did but would have difficulty now), 2 (requires assistance), 3 (dependent). Total scores range from 0 to 33, higher scores indicates a higher functional impairment.^37^

### 2.5 Dementia severity

CDR^14^ was used to evaluate six key domains: memory, orientation, judgment and problem‐solving, community affairs, home and hobbies, and personal care. It uses a scoring system ranging from 0 (no dementia) to 3.0 (severe dementia). We used both the CDR‐GS and the CDR‐SB as scoring methods for the CDR. The CDR‐GS is a commonly scoring method based on comprehensive and dementia assessments. CDR‐GS is mainly based on memory and integrates five minor areas to classify the severity of dementia into five stages: normal (0 points), suspected dementia (0.5 points), mild dementia (1 point), moderate dementia (2 points), and severe dementia (3 points) according to the scoring criteria. CDR‐SB is a comprehensive evaluation of six different cognitive areas, with a maximum score of 3 points for each cognitive area, and a scoring range of 0–18 points for the overall score.^38^ Every rater (R.M., G.V., P.B., and M.N.‐H.) obtained training and was certified by the ReDLat consortium.^37^ The raters apply a semi‐structured interview of CDR to both participant and informant; then, a data entry registers every domain's answer to an ad‐hoc platform on the website to obtain total CDR‐GS from 0 to 3. The scores for each domain are then entered into an Excel to obtain the CDR‐SB ranging from 0 to 18.

### 2.6 CDR cultural adaptations

We administered the Spanish version of CDR, used by the ReDLat consortium, which develops strategies for harmonization of participant enrolment across sites, including adaptation and validation.^37^ The Peruvian version of CDR included changes to adapt it to local culture and society, while maintaining the rigor of the questions. For example, in the calculation section, the question “How many nickels are in a dollar?” was replaced with “¿Cuántas monedas de 20 céntimos hay en 1 sol?” The correct answer is that one Peruvian sol is equivalent to five 20‐cent coins. In Peru, 1 sol equals 100 cents (See Table 1).

**TABLE 1:** Cultural adaptations of CDR, from the original version and the Spanish version to the Peruvian version.

Clinical dementia rating	Memory	Orientation	Judgment and problem solving	Community participation	Housework and hobbies	Personal care
**Original version (English)**	**CAREGIVER or INFORMANT**: Question 12 item 3 “Highest level achieved”	**CAREGIVER or INFORMANT**: No changes were made in this section	**CAREGIVER or INFORMANT**: No changes were made in this section	**CAREGIVER or INFORMANT**: No changes were made in this section	**CAREGIVER or INFORMANT**: No changes were made in this section	**CAREGIVER or INFORMANT**: No changes were made in this section
	**SUBJECT**: Question 3: John Brown, 42, Queen Street, Auckland **SUBJECT**: Question 6: “Highest level achieved” **SUBJECT**: Question 10: John Brown, 42, Queen Street, Auckland	**SUBJECT**: No changes were made in this section	**SUBJECT**: Question 5: How many 5‐cent pieces are in a dollar? **SUBJECT**: Question 6: How many 20 cent pieces in a 5.40 dollar?	**Not applicable** (these sections only correspond to the caregiver or informant)		
**ReDLat Consortium version (Spanish)**	**CAREGIVER or INFORMANT**: Question 12 item 3 “Curso”	**CAREGIVER or INFORMANT**: No changes were made in this section	**CAREGIVER or INFORMANT**: No changes were made in this section	**CAREGIVER or INFORMANT**: No changes were made in this section	**CAREGIVER or INFORMANT**: No changes were made in this section	**CAREGIVER or INFORMANT**: No changes were made in this section
	**SUBJECT**: Question 3: Juan García, Calle Aragón, 42, Barcelona **SUBJECT**: Question 6: “Curso” **SUBJECT**: Question 10: Juan García, Calle Aragón, 42, Barcelona	**SUBJECT**: No changes were made in this section	**SUBJECT**: Question 5: ¿Cuántas pesetas hay en un duro? **SUBJECT**: Question 6: ¿Cuántos duros se necesitan para tener 135 pesetas?	**Not applicable** (these sections only correspond to the caregiver or informant)		
**Peru's versión (Spanish)**	**CAREGIVER or INFORMANT**: Question 12 item 3 “Estudios realizados” (cultural)	**CAREGIVER or INFORMANT**: No changes were made in this section	**CAREGIVER or INFORMANT**: No changes were made in this section	**CAREGIVER or INFORMANT**: No changes were made in this section	**CAREGIVER or INFORMANT**: No changes were made in this section	**CAREGIVER or INFORMANT**: No changes were made in this section
	**SUBJECT**: Question 3: Juan Quispe, Avenida Arequipa, 420, Lima **SUBJECT**: Question 6: “Estudios realizados” (cultural) **SUBJECT**: Question 10: Juan Quispe, Avenida Arequipa, 420, Lima	**SUBJECT**: No changes were made in this section	**SUBJECT**: Question 5: ¿Cuántas monedas de 5 centavos hay en 1 sol? **SUBJECT**: Question 6: ¿Cuántas monedas de 20 centavos hay en 5.40 soles?	**Not applicable** (these sections only correspond to the caregiver or informant)		

Abbreviation: CDR, Clinical Dementia Rating scale.

### 2.7 Data analysis

The variables of interest for these analyses are the correspondence between the gold standard—the diagnosis given by a multidisciplinary team of health professionals (notably neuropsychologists, neuro‐rehabilitators, geriatricians and neurologists)—and the CDR results. The number of true positives, true negatives, false positives, and false negatives represents this correspondence. These values are of primary importance when evaluating the discriminative validity of the CDR, including sensitivity, specificity, positive and negative predictive values, and the Youden index. To assess test reliability of the CDR, Cronbach's alpha will be calculated. To assess the concurrent validity of the CDR, correlations among the MMSE, RUDAS, PFAQ, and UDS components will be examined. The CDR‐GS classification will also be analyzed using ANOVA (CDR classification × neuropsychological tests) to assess its ability to classify participants and to determine whether it differs, in score, from other tests of interest in dementia.

If the conditions for parametric analyses are not met, nonparametric alternatives will be applied to the data, using the same variables under the same conditions. The following analyses will be partly performed using the JASP program from the University of Amsterdam (JASP 0.18.3, Apple Silicon version).

### 2.8 Ethics statement

The study was approved by the Committee for Medical and Health Research Ethics, Hospital Nacional Docente Madre‐Niño‐ HONADOMANI “San Bartolomé” (12360‐17). Written informed consent was obtained from all patients and informants enrolled in the study, and was conducted in accordance with the Declaration of Helsinki. Cognitively healthy participants and those with MCI provided written informed consent. Participants with early‐stage and moderate AD also provided informed consent, supplemented by the signatures of their respective informants. In all cases, two copies of the informed consent form were signed: one was provided to the participant, while the other was archived in the participant's file.

## 3 RESULTS

Table 2 presents the demographic data for the study population, including sample size, age, sex, years of education, and scores on the following tests: CDR‐SB, MMSE, PFAQ, MoCA, and RUDAS. Data are presented as the CDR‐GS classification: 0, 0.5, and ≥1.0, of 902, 271, and 427 participants, respectively. A chi‐squared test was performed to compare sex distributions across the CDR groups, but it did not yield statistically significant differences. The mean age of each CDR‐GS group was 65.74 ± 7.83, 72.64 ± 8.20, and 75.95 ± 8.37, respectively. An analysis of variance (ANOVA) revealed statistically significant differences between the CDR groups on age (*p* < 0,001, ω^2^ = 0.24, CI = [0.21, 0.28]) and on each of the following tests: MMSE (*p* < 0.001, ω^2^ = 0.41, CI = [0.36, 0.47]), PFAQ (*p* < 0.001, ω^2^ = 0.64, CI = [0.61, 0.67]), MoCA (*p* < 0.001,  ω^2^ = 0.66, CI = [0.61, 0.71]), and RUDAS (*p* < 0.001, ω^2^ = 0.53 CI = [0.49, 0.58]).

**TABLE 2:** Demographic data of participants in each diagnostic group according to CDR classification (*N* = 1600).

Demographic data	CDR‐GS = 0	CDR‐GS = 0.5	CDR‐GS ≥ 1	Total sample	Test
Gender (F/M)	640/262	178/93	308/119	1126/474	Chi‐square
Age	65.74 ± 7.83	72.64 ± 8.28	75.95 ± 8.37	69.63 ± 9.25	ANOVA
Education	11.47 ± 6.06	8.56 ± 6.45	10.42 ± 5.62	10.70 ± 6.11	ANOVA
CDR‐SB	0.01 ± 0.24	1.47 ± 1.121	7.99 ± 4.16	2.39 ± 4.07	ANOVA
MMSE	26.62 ± 3.61	24.87 ± 3.65	18.38 ± 5.79	24.49 ± 5.56	ANOVA
PFAQ	0.25 ± 1.38	2.80 ± 4.39	16.48 ± 9.20	5.17 ± 8.79	ANOVA
MoCA	23.91 ± 3.51	18.10 ± 4.89	9.60 ± 6.07	17.12 ± 8.43	ANOVA
RUDAS	26.37 ± 2.62	23.18 ± 3.63	15.04 ± 7.65	22.65 ± 7.09	ANOVA

*Note*: Analyses of variance between the healthy control group (CDR‐GS 0) vs the mild cognitive impairment group (CDR‐GS 0.5), and the Alzheimer's disease group (CDR‐GS ≥1). Mean ± standard deviation. Analyses of variance between CDR‐GS groups.

Abbreviations: AD, Alzheimer's disease; C, control; CDR‐GS, Clinical Dementia Rating scale‐Global Score; CDR‐SB, Clinical Dementia Rating Sum of Boxes score; MCI, mild cognitive impairment; MMSE, Mini‐Mental State Examination; MoCA, Montreal Cognitive Assessment; PFAQ, Pfeffer Functional Activity Questionnaire; RUDAS, Rowland Universal Dementia Assessment.

### 3.1 Internal consistency

Regarding the reliability evaluation of the CDR, a Cronbach's alpha inter‐item analysis was performed based on the six domain scores (memory, orientation, judgment and problem solving, community affairs, home and hobbies, personal care, and language) revealing high internal consistency between domains: *α* = 0.91 (95% CI: 0.91–0.92) for the total sample, an *α* = 0.81 (95% CI: 0.79–0.83) for the control group, *α* = 0.70 (95% CI: 0.64–0.75) for the MCI group, and *α* = 0.89 (95% CI: 0.88–0.89) for the AD group. All these results are presented in Table 3 and are statistically significant (*p* < 0.01).

**TABLE 3:** Cronbach's alpha if item dropped and item‐rest correlation for each CDR domain.

Domains	“If item dropped” Cronbach's *α*	“Item‐rest” correlation
Memory	0.968	0.900
Orientation	0.966	0.921
Judgment and problem solving	0.964	0.936
Community participation	0.963	0.949
Housework and hobbies	0.965	0.929
Personal care	0.975	0.830

*Note*: Frequentist individual domain reliability statistics on 1600 participants. Item‐rest correlation refers to the correlation between each item and the total score computed without that item (domain). Cronbach's alpha if item dropped indicates the internal consistency that would be obtained if the item were removed.

Abbreviation: CDR, Clinical Dementia Rating scale.

### 3.2 Discriminant validity

To evaluate the CDR efficacy, the participants’ diagnoses were assigned to each CDR classification score: a CDR‐GS of 0.5 is associated with a MCI diagnosis, a CDR‐GS of ≥ 1 is associated with dementia, and a CDR‐GS of 0 is associated with healthy control participants. All results of univariate receiver operating characteristics curve (ROC) on CDR effectiveness in screening for MCI participants and AD patients are presented in Table 4. These results led to the identification of an optimal CDR‐SB cutoff score of 0.5 for detecting MCI relative to healthy controls and a cutoff score of 1 for detecting AD relative to healthy controls.

**TABLE 4:** Effectiveness of the CDR in screening for MCI and AD patients.

Diagnostic performance	MCI/NC		AD/NC	
	CDR‐SB	CDR‐GS	CDR‐SB	CDR‐GS
AUC	98.5	99.80	99.90	99.90
Accuracy	99.39	99.74	99.11	99.93
Sensitivity	92.59	100	98.21	99.77
Specificity	100	99.67	99.56	100
Positive predictive	100	98.80	99.09	100
Negative predictive	99.34	100	99.12	99.89
Youden's index	92.59	99.70	97.80	99.77

*Note*: Values of reliability assessment components when comparing the MCI group and the control group, and between the AD group and the control group (% values). AUC values shown to be significant for each comparison.

Abbreviations: AD, Alzheimer's disease; AUC, area under the curve; CDR‐GS, Clinical Dementia Rating scale Global Score; CDR‐SB, Clinical Dementia Rating Sum of Boxes score; MCI, mild cognitive impairment; NC, normal cognition.

### 3.3 Convergent validity

**FIGURE 2:**
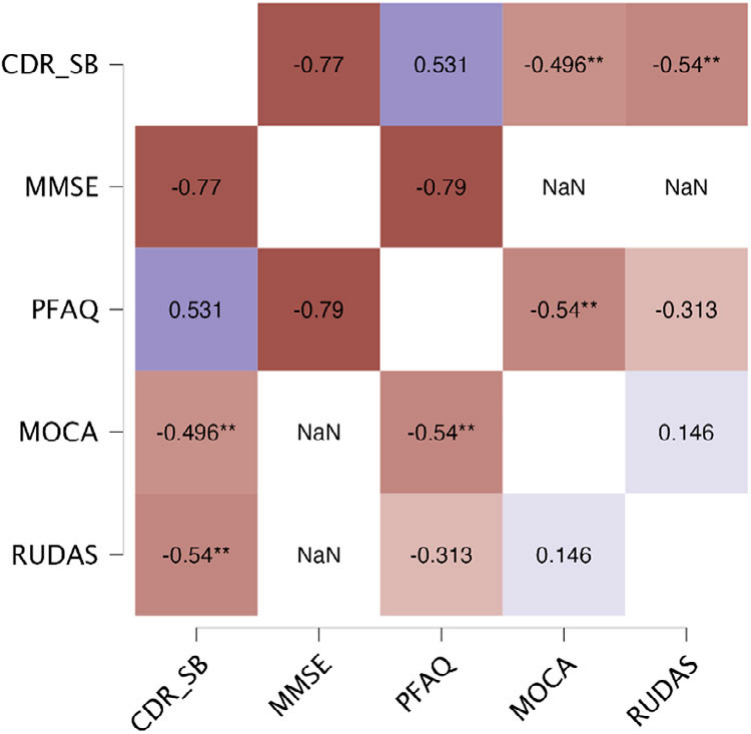
Partial Spearman's rho heatmap: correlations between the CDR sum of boxes scoring method and the main cognitive and activity assessments for the MCI group. CDR‐SB, Clinical Dementia Rating Sum of Boxes scoring method (0–18); MCI, mild cognitive impairment; MMSE, Mini‐Mental State Examination (0–30); MoCA, Montreal cognitive assessment (0–30); PFAQ, Pfeffer Functional Activities Questionnaire (0–33); RUDAS, Rowland Universal Dementia Assessment Scale (0–30). **, < 0.01.

**FIGURE 3:**
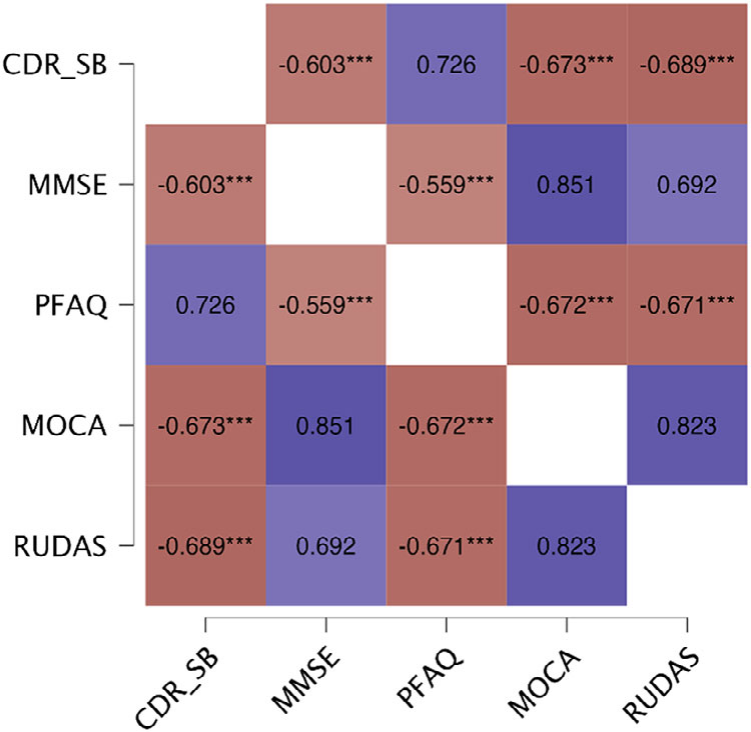
Partial Spearman's rho heatmap: correlations between the CDR‐SB scoring method and the main cognitive and activity assessments for the AD group. AD, Alzheimer's disease; CDR‐SB, Clinical Dementia Rating Sum of Boxes scoring method (0–18); MMSE, Mini‐Mental State Examination (0–30); MoCA, Montreal Cognitive Assessment (0–30); PFAQ, Pfeffer Functional Activities Questionnaire (0–33); RUDAS, Rowland Universal Dementia Assessment Scale (0–30). ***, < 0.01.

To assess the convergent validity of the CDR with other neuropsychological tests, Kendall's correlations were performed on the CDR‐GS and Spearman's correlations were performed on the CDR‐SB with the MMSE, the PFAQ, the MoCA and all of the UDS components performance: digit span in order, digit span in inversed order, the semantic fluency, the TMT‐B, the Craft Story 21, the Benson figure, Multilingual Naming Test (MINT), and the phonological fluency. Overall, the healthy control group's performance on the CDR showed no statistically significant correlations with the other tests, except for the PFAQ (tau b = 0.10, *p* < 0.01) and the TMT‐B score (tau b = 0.08, *p* < 0.01). Regarding the CDR, no correlations could be computed for the healthy control group because all healthy participants scored 0. This floor effect is expected in normal aging on the CDR test and explains why the clinical scales do not correlate with neuropsychological measures in this specific population. The MCI group's performance showed a statistically significant correlation between the CDR score and the other tests (*r* = −0.50 to −0.70; *p* < 0.01), except for the PFAQ, MMSE, and digit span scores when considering the CDR SB scoring method (Figure 2). For the AD group, the CDR‐GS and CDR‐SB scores correlated statistically significantly (correlations range: |tau b| = 0.23–0.57; |r| = 0.34–0.69; *p* < 0.01) with all other tests of interest except for the PFAQ. The CDR‐GS and the CDR‐SB scores also correlated with them |tau b| = 0.22–0.64; |r| = 0.25–0.81; *p* < 0.01) except for the PFAQ (Figure 3). All correlations are corrected for age and years of education to control for external variables when assessing convergent validity. All the results of the validity analysis of the CDR are presented in Table 5. Post‐hoc power analyses (using the pwr package in R) confirmed adequate statistical power for key comparisons in smaller subgroups. Specifically, with *n* = 271 (MCI), power was 87% to detect moderate rank correlations (τ = 0.25) and ∼100% to detect clinically meaningful diagnostic accuracy (area under the curve [AUC] = 0.80; *ρ* = 0.60). In the AD subgroup (*n* = 427), power exceeded 97% for correlations and was essentially 100% for AUC = 0.80 (G*Power 3.1/pwr R package). When assessing discriminant validity and convergent validity for the CDR, no data were missing.

**TABLE 5:** CDR convergent validity between cognitive and functional evaluation with each diagnostic group according to CDR scoring.

Cognitive and functional briefly and neuropsychological comprehensive evaluation	NC group *N* = 902		MCI group *N* = 271		AD group *N* = 427		Total sample *N* = 1600	
	CDR		CDR		CDR		CDR	
	GS	SB	GS	SB	GS	SB	GS	SB
MMSE	−0.031	/	−0.825^*^	−0.77	−0.453^***^	−0.603^***^	−0.546^***^	−0.68^***^
PFAQ	0.103^***^	/	0.061	0.531	0.573^***^	0.726	0.736	0.811^***^
MoCA	0.024	/	−0.326^*^	−0.496^**^	−0.538^***^	−0.673^***^	−0.643^***^	−0.769^***^
RUDAS	0.004	/	0.037	−0.54^**^	−0.535^***^	−0.689^***^	−0.633^***^	−0.765^***^
Digit span in order	−0.026	/	/	−0.049	−0.3^***^	−0.344^***^	−0.218^***^	−0.25^***^
Digit span in inverse order	−0.042	/	/	−0.095	−0.228^***^	−0.419^***^	−0.344^***^	−0.402^***^
Semantic fluency	−0.001	/	/	−0.703^***^	−0.386^***^	−0.524^***^	−0.417^***^	−0.5^***^
Trail Making Test, part B	0.077^*^	/	/	0.556	0.267^**^	0.457	0.298	0.361^***^
Craft story 21	−0.026	/	/	−0.553^**^	−0.25^***^	−0.303^***^	−0.453^***^	−0.55^***^
Benson complex figure copy	−0.003	/	/	−0.551^**^	−0.276^***^	0.415^***^	−0.43^***^	−0.503^***^
Multilingual Naming Test	−0.078	/	/	−0.622^***^	−0.305^***^	−0.449^***^	−0.38^***^	−0.449^***^
Phonological fluency	0.01	/	/	−0.031	−0.489^***^	−0.66^***^	−0.394^***^	−0.503^***^

*Note*: Values are correlations’ values of Spearman's *r* for CDR‐SB scores and Kendall's tau b for CDR‐GS.

Abbreviations: AD, Alzheimer's disease; C, control; CDR‐GS, Clinical Dementia Rating scale Global Score; CDR‐SB, Clinical Dementia Rating Sum of Boxes score; MCI, mild cognitive impairment; MMSE, mini‐mental state examination; MoCA, Montreal cognitive assessment; NC, normal cognition; PFAQ, Pfeffer functional activity questionnaire; RUDAS, Rowland universal dementia assessment.

* Indicates a *p*‐value equal or less than 0.05.

** Indicates a *p*‐value equal to or less than 0.01.

*** indicates a *p*‐value equal or less than 0.001.

## 4 DISCUSSION

The Peruvian's CDR demonstrated good psychometric properties and the results were comparable to those obtained in CDR evaluation of LA.^39^, ^40^ Although the CDR is widely used in LA, it has been evaluated among population from clinical settings^41^; therefore, it is important understand its effectiveness in screening for MCI and dementia among community‐dwelling older people.

In this study, CDR exhibits excellent internal consistency across subgroups. This finding is consistent with studies conducted by McDougall et al.,^41^ and Nguyen et al.,^42^ supporting CDR's internal consistency among clinic‐based population. Additionally, the study found that when screening for MCI, the sensitivity and specificity of the CDR‐SB scoring and the CDR‐GS scoring method were very close (92.5%, 100%) and (100%, 99.7%), respectively. When screening for AD, the CDR‐SB had high sensitivity (98.2%) and specificity (99.6%), whereas CDR‐GS had relatively high sensitivity (99.8%) and excellent specificity. The Vietnamese version of CDR‐GS for detecting dementia showed sensitivity of 93.6% and ideal specificity^43^; meanwhile, in the Brazilian's CDR detection of dementia among healthy elderly or MCI was 86% and 80% sensitive, respectively, and 100% specific for both settings.^41^ The high ROC indices should be interpreted cautiously: participants were part of a specialized care setting. This context may contribute to the inflation of psychometric properties, overestimating diagnostic performance in comparison to a broader community care population.

Some studies have shown that CDR‐SB is more sensitive and can better detect early‐stage changes in cognitive function^44^ and at classifying the severity of cognitive impairment in patients with low educational attainment.^44^ CDR is a multi‐dimensional measure of intra‐individual decline in cognition, behavior, and function.^14^ It thus minimizes confounding factors such as age, sex, literacy, ethnicity, and culture. When dementia is present, the CDR monitors its entire course from very mild to severe, avoiding floor and ceiling effects. CDR‐GS assesses changes in global cognitive function, whereas CDR‐SB evaluates performance across cognitive domains. Therefore, we recommend choosing the appropriate scoring method based on the patient's specific situation. If an evaluation of overall cognitive function is needed, CDR‐GS may be the preferred method. If early detection of cognitive impairment or evaluating treatment effects is needed, CDR‐SB may be more suitable.^43^ In Peru, clinicians may use the CDR‐SB scoring to follow‐up patient with MCI and detect conversion to mild AD; meanwhile, a neuropsychologist may prefer using the CDR‐GS scoring to classify dementia stage.

Our findings show a high correlation between CDR‐GS and CDR‐SB scoring with the total scores of BCSs and BFSs tools. The correlations from the AD group are consistent with those of the overall sample, except to PFAQ, demonstrating CDR's effectiveness in reflecting the severity of subjective and objective cognitive impairment.^43^ At the expense of lower amplitude values, the CDR‐SB scores have the advantage of greater accuracy in identifying early stages of dementia, monitoring disease progression, and classifying dementia severity relative to the CDR‐GS.^6^, ^45^ However, in the MCI sample, CDR scores correlate less strongly with total scores on MoCA and RUDAS. This indicates that the effectiveness of CDR in reflecting the severity of cognitive impairment is relatively diminished in the MCI.^46^ The low correlation between CDR and PFAQ in the MCI and AD groups is noteworthy, in contrast to previous studies.^47^ A likely explanation could be related to the clinical judgment synthesized by the experienced clinician in comparison of the self‐reported complaints aspects of the PFAQ. The time clinicians have to conduct two interviews, so a trained clinician synthesizes all available information from the participant and the study partner to determine the domain box score. However, it is unclear to what extent and how each question affects the box score, and whether specific questions are better at diagnosing dementia status than others. Furthermore, there may be substantial variability of dementia severity within the same levels of a CDR‐GS.^48^, ^49^ In this sense, activities of daily living (ADL) correlation with the CDR‐SB was slightly lower across all time points, indicating a weak correlation between the CDR‐SB and informant‐reported outcomes of ADLs compared with cognitive performance‐based measures.^50^ Therefore, conceptually, we consider the cognitive measures to be more aligned with the CDR‐SB. Second, changes in ADLs may be more readily observable and reliably reported by care partners. In contrast memory changes may be more noticeable and robustly quantifiable through cognitive assessments performed by trained clinicians. Of note, estimates for the memory anchor were similar, with slightly greater support for the lower end of the threshold range.^51^

### 4.1 Clinical implications

In this Peruvian sample, both CDR‐GS and CDR‐SB showed excellent ability to discriminate between controls, MCI, and AD, suggesting that they can be confidently integrated into routine clinical decision‐making. These results have implications for diagnostic pathways in LA contexts. Given its informant‐based structure and limited dependence on formal education, the CDR can contribute to an evaluation of cognitive and functional decline, taking into account the literacy context of Peru. Future work could examine the performance of the Peruvian CDR in primary care, as well as its longitudinal responsiveness to clinically meaningful change in MCI and early AD; however, it requires specialized certification, an experienced clinician and at least 1 hour to complete it; therefore, its applicability to public health services is limited in Peru.

### 4.2 Strengths and limitations

The first strength is the inclusion of a well‐characterized sample of 1600 participants covering the full spectrum from cognitively healthy to MCI and AD, which enhances the precision and the robustness of psychometric estimates. Second, the diagnostic classification was based on a multidisciplinary consensus, neuropsychological and complementary investigations, providing a rigorous gold standard against which to evaluate CDR performance. Third, convergent validity was assessed using tools previously validated in Peru. However, several limitations should be acknowledged. The diagnostic performance observed in this study should be interpreted cautiously because the group assignment partly relied on the CDR classes. Therefore, rather than providing an independent test of diagnostic accuracy, our findings mainly support the clinical applicability of the Spanish version of the CDR in this Peruvian clinic‐based setting. While our sample was balanced by education, it was predominantly female, and it was limited to an urban population, potentially reducing generalizability to rural areas where education and gender disparities and dialectical variations may be more pronounced in our country. A priori, gendered caregiving roles and women over‐representation may influence informant reports, but female caregivers who were married were less likely to experience burden possibly due to support from their spouses in caregiving responsibilities explained by higher perceived social support.^52^ Additionally, the majority of family caregivers (88%) who experienced little to no caregiver burden were attributed to cultural factors such as filial piety and responsibilities. Similar findings were reported in Spain, which confirms the protective role of positive mental health^50^; however, we did not find similar reports in Latin American studies. Future studies should expand to include a multicenter studies from rural contexts, representing a more diverse group of patient–caregiver pairs.^53^ Second, no biological biomarkers on CSF or PET were evaluated, limiting the confirmation of underlying neuropathology,^54^ but recent reports in the native Peruvian population suggest that plasma pTau‐217 showed significant correlations with RUDAS and PFAQ to supplement the AD diagnostic process when other precise instruments are not available/feasible.^7^ Having an accurate and appropriate strategy for early detection of dementia is crucial in LA: reaching consensus on diagnosis based on adapted BCS and BFS tools; supported by neuropsychological evaluation, CDR, neuro‐imagens and biomarker available in every country, but the pendent task include analysis of correlation between CDR‐SB with BBBs for primary care. For future adequate use of disease‐modifying therapies in LA, specialists need to harmonize the NIA‐AA diagnostic criteria based on local availability of BBBs with adequate screening to select participants, ideal staging to randomize them and provide follow‐up and adherence with validated tools and we believe that CDR‐SB meets these requirements. Another limitation is that we did not take into account caregiver burden and depression measured in detail, that could alter the responses to the questionnaires related to ADL and CDR, but they were excluded if they had alterations on the Zarit scale and PHQ‐9. However, low correlations have previously been shown between CDR with caregiver burden and depression, which suggests these potential confounders would have little to no influence.^55^ Finally, our oldest adult was 79 years of age, so future studies should extend to adults 80+ years of age, who in geriatric and memory clinics often show mixed pathologies, sensory loss, and frailty.

## 5 Conclusions

The present study demonstrated the clinical validity of the CDR for classifying dementia severity in a Peruvian clinic‐based population and suggests that this Spanish version may be appropriately applied in this research setting, and provides evidence for two different scoring methods. These findings provide a basis for further research on the growing population of the elderly in Peru, an understudied ethnically and culturally diverse country, focused on further understanding risk factors leading to dementia and its stage progression.

## AUTHOR CONTRIBUTIONS

Katherine Agüero: conceptualization, critical revision, final approval. Maria Fe Albujar‐Pereyra: methodology, critical revision, final approval. Pamela Bartolo: conceptualization, critical revision, final approval. Belen Custodio: methodology, analysis, writing, critical revision, final approval. Nilton Custodio: conceptualization, methodology, analysis, writing, critical revision, final approval. José Huilca: conceptualization, critical revision, final approval. Rosa Montesinos: conceptualization, writing, critical revision, final approval. Milagros Nuñez‐Huanca: methodology, critical revision, final approval. Junior Senador: conceptualization, methodology, analysis, writing, critical revision, final approval. Graciet Verastegui: conceptualization, critical revision, final approval.

## CONFLICT OF INTEREST STATEMENT

Nilton Custodio and Rosa Montesinos are partially supported by the following NIH grants: AG057234, R56AG069118‐01, SG‐21‐715176‐LATAM FIN‐. Author disclosures are available in the supporting information. GERS, 24AARG‐D‐1246942. Apart from that, the authors declare that they have no conflicts of interest related to the research, authorship, and/or publication of this article. Author disclosures are available in the Supporting Information.

## CONSENT STATEMENT

The study was approved by the Committee for Medical and Health Research Ethics, Hospital Nacional Docente Madre‐Niño‐ HONADOMANI “San Bartolomé” (12360‐18). Written informed consent was obtained from all patients and informants enrolled in the study, and was conducted in accordance with the Declaration of Helsinki.

## Supporting information




Supporting Information

## Data Availability

The authors are willing to allow the journal to review their data if requested.
